# Prediction of immunotherapeutic responses by a classifier model based on inflammation-associated tumor microenvironment signatures in colorectal cancer

**DOI:** 10.1007/s12672-026-04548-6

**Published:** 2026-02-01

**Authors:** Ziqi Gong, Yuxian Feng, Jing Tu

**Affiliations:** State Key Laboratory of Digital Medical Engineering, School of Biological Science and Medical Engineering, Southeast University, Nanjing, 210096 China

**Keywords:** Single cell RNA sequencing, Immunotherapy, Inflammation, Classifier model, Colorectal cancer

## Abstract

**Background:**

In recent years, the application of immunotherapy has greatly improved the prognosis of cancer patients. However, a proportion of patients will acquire resistance to immunotherapy, leading to a lower response rate and poorer clinical outcome. The underlying mechanisms contributing to the therapeutic resistance and accurate biomarkers to predict immunotherapy responses remain unclear.

**Methods:**

We comprehensively analyzed a single cell RNA-sequencing dataset of microsatellite instability-high colorectal cancer patients received anti-PD1 immunotherapy. We dissected the heterogeneity of the immunosuppressive tumor microenvironment contributing to the therapeutic resistance and highlighted on a correlation between pro-inflammatory factors and inhibited immune responses. We established a classifier model using Random Forest algorithm based on the common marker genes of inflammation-associated subpopulations. The validation of the model and further analysis between potential responders and non-responders was also performed in bulk RNA-seq cohorts.

**Results:**

Three inflammation-related cell subgroups, including CEMIP+ Monocytes, CCL4 + Neutrophils and MMP3 + Fibroblasts were identified to be associated with immune-suppressed signatures and unfavorable responses to immunotherapy. The classifier model based on inflammatory signatures exhibited acceptable accuracy and robustness to predict immunotherapeutic responses across cancer types.

**Conclusion:**

Our study dissected the heterogeneity of the immunosuppressive tumor microenvironment and highlighted a correlation between pro-inflammation signatures and inhibited anti-tumor immunity. We also developed a novel classifier model based on inflammation-related signatures to predict patients’ responses to immunotherapy.

**Supplementary Information:**

The online version contains supplementary material available at 10.1007/s12672-026-04548-6.

## Introduction

Colorectal cancer (CRC) is a common malignancy of gastrointestinal system with rising morbidity, which ranks as the second leading cause of cancer-related death globally [1]. For many patients with advanced diseases, conventional treatment strategies including surgical resection, radiotherapy and chemotherapy are not satisfactory [2]. In the last few years, the prognosis of CRC patients has been greatly improved due to the rapid development and widely application of immunotherapy [3, 4]. FDA has proved programmed death-1 (PD-1) inhibitor as a first-line therapy for patients with metastatic microsatellite instability-high (MSI-H) CRC [5–8]. Despite the benefits, a proportion of patients will develop into refractory to immunotherapy. The potential mechanisms contributing to the failure of immune checkpoint inhibitor (ICI) immunotherapy and effective biomarkers to predict the therapeutic responses remain unclear [9].

Emerging evidences from high-throughput sequencing have revealed the important roles of tumor microenvironment (TME), a complicated network made up of tumor cells, stromal cells and immune cells in cancer development and therapeutic resistance [10, 11]. Previous researches have reported the interactions among cells in TME and identified potential targets to enhance the efficacy of immunotherapy [12, 13]. However, traditional bulk sequencing was insufficient to analyze the heterogeneity of cells, where the expression levels of all cells were averaged and the unique properties of individual cell were obscured. Nowadays, single cell RNA sequencing (scRNA-seq) has brought us a powerful technology to dissect distinct characteristics of transcriptomic profiles at single cell resolution, promoting our understanding of tumor heterogeneity and molecular mechanisms leading to different clinical responses [14–17]. Several studies have described the complexity of CRC microenvironment and investigated novel therapeutic approaches targeting specific cell types [18–20]. In recent years, advances in potential mechanisms mediating immunotherapeutic resistance have also been discussed. A study of repair-deficient (d-MMR)/MSI-H CRC revealed a correlation between proinflammatory molecules and immunosuppressive TME [21]. Another research found that an interferon-high immunophenotype enriched in cytotoxic lymphocytes and antigen-presenting macrophages could identify clinical benefit from immunotherapy for CRC patients [22]. However, few studies focused on the comprehensive investigation on the predictive value of inflammation-associated TME signatures to evaluate the clinical efficacy of immunotherapy, which supposed to be a prerequisite for improving precise therapeutic strategies and clinical stratification.

The integrated analysis of scRNA-seq and bulk RNA-seq allows researchers to have a comprehensive view of overall gene expression patterns both at the tissue and single-cell levels to discover effective biomarkers, which could enhance clinical classification and the development of more effective therapeutic strategies. In this study, based on scRNA-seq data from a published literature, we comprehensively investigated on the cell type distributions and functional signatures in immunotherapy-resistant and sensitive CRC patients and elucidated a correlation between pro-inflammatory and immunosuppressive TME. Pro-inflammatory factors from specific subgroups of immune cells and stromal cells were highly expressed in immunotherapy-resistant patients. Based on cell signatures of the inflammatory TME, we constructed a random forest model to stratify patients’ responses to immunotherapy.

## Methods

### Data collection and preprocessing

#### Single cell RNA-seq data

We comprehensively searched public datasets for scRNA-seq data involving information about the responses to ICI immunotherapy of CRC patients. Samples from 6 patients with high-MSI CRC who received 3-week cycle of anti-PD-1 immunotherapy were included in the study (PRJNA932556) [23]. At the end of the treatment, radiographic evaluation determined disease remission in 3 samples (S1, S2, and S3) which were defined as sensitivity to immunotherapy (S). The other 3 samples (R1, R2, and R3) were considered to be resistant to the therapy (R), which showed a progression in both primary and metastatic lesions. Raw data of scRNA-seq generated by 10X Genomics platform was obtained from The European Nucleotide Archive (ENA) database (https://www.ebi.ac.uk/ena/browser/home). “Bamtofastq” function in 10X Genomics Cell Ranger software (version 7.2.0) was applied to convert BAM files to FASTQ files. Then we used “count” pipeline to demultiplex, align FASTQ files to human reference genome (https://cf.10xgenomics.com/supp/cell-exp/refdata-gex-GRCh38-2020-A.tar.gz) and generate the gene-cell unique molecular identifier (UMI) matrix.

#### Bulk RNA-seq data

We also included bulk RNA-seq datasets from TCGA database and three ICI cohorts (IMvigor210, GSE78220 and GSE126044) to validate the results inferred from single cell analysis and develop a classifier model for the prediction of immunotherapeutic responses across cancer types. Transcriptome data and clinical information of 524 colon cancer and 177 rectal cancer samples from TCGA-COAD and TCGA-READ cohort were obtained using easyTCGA package (version 0.0.4) in R software (version 4.1.3) and merged together as TCGA-CRC cohort. The TPM matrix was transformed by log2(TPM + 1). IMvigor 210 was a phase 2 single arm study evaluating the efficacy of PD-L1 inhibitor atezolizumab for metastatic urothelial cancer patients. We accessed the expression and clinical information of 298 patients in IMvgior210 cohort using R package IMvigor210CoreBiologies. The expression matrix and therapeutic responses information from GSE78220 cohort for 27 melanoma patients and GSE126044 cohort for 16 non-small cell lung cancer (NSCLC) patients were downloaded from GEO database (https://www.ncbi.nlm.nih.gov/geo/). Patients from IMvgior210 and GSE126044 were divided into “Response”/R and “Non-response”/NR. Patients in GSE78220 dataset were categorized into Complete Response (CR), Partial Response (PR) and Progressive Disease (PD) according to clinical evaluation. We defined CR and PR as “Response” and PD as “Non-response”.

Among the bulk RNA-seq datasets included in our study, IMvigor210 cohort and two datasets from GEO database (GSE78220 and GSE126044) are immunotherapy-related cohorts containing information of patients’ therapeutic responses. IMvigor210 was used as the training set for constructing our predictive classifier. GSE78220 and GSE126044 served as external validation sets. In addition, IMvigor210 cohort was also used to validate the association between gene expression features of CEMIP+ Monocytes and MMP9 + Fibroblasts and patients’ response to immunotherapy. GSE78220 was used to validate the expression characteristics of CCL4 + Neutrophils in relation to immunotherapy outcomes. We also incorporated TCGA-COAD and TCGA-READ datasets to investigate CRC expression profiles. Expression data for all datasets were downloaded or transformed into TPM format. The immunotherapy datasets were analyzed individually without batch effect correction or additional normalization. For TCGA-COAD and TCGA-READ cohorts, we merged the TPM expression matrices based on shared genes. Since principal component analysis (PCA) showed no significant batch effects between the two cohorts, batch correction was not performed.

#### Quality control of ScRNA data and across-sample integration

Seurat package (version 4.3.0) was used to analyze the expression matrix of scRNA-seq [24]. We first filtered out cells with fewer than 200 detected genes or genes expressed in fewer than 3 cells. To further remove low-quality cells, we filtered cells by removing those expressing < 300 or > 8,000 genes and harboring high mitochondrial gene expression (≥ 75%). For the percentage of mitochondrial gene expression, we applied an adjusted threshold of 75%, as a considerable subset of epithelial cells exhibited high mitochondrial gene expression (Figure S1A-B). This threshold was chosen to avoid discarding metabolically active cells with biological relevance especially for malignant phenotype, as supported by previous reports [21, 25]. We normalized gene expression to eliminate the effect of sequencing depth and selected top 2,000 hypervariable genes by “NormalizeData” and “FindVariableFeatures” functions respectively (Figure S1C). PCA was performed to choose the most important 30 components for further integration (Figure S1D) and clustering after scaling the data and regressing out covariates like the expression of mitochondrial gene and cell-cycle scores. To integrate cells from different samples and remove batch effects, package harmony (version 1.2.0) was applied based on the results of PCA [26].

### Dimension reduction and unsupervised clustering

We used the uniform manifold approximation and projection (UMAP) method for the visualization of dimension reduction analysis with “RunUMAP” function in Seurat [27]. “FindNeighbours” and “FindClusters” functions were performed subsequently to identify different cell clusters through unsupervised clustering using Louvain algorithm. The optimal clustering resolution was determined using clustree package (version 0.5.1), which visualized cluster transitions across resolutions. Based on this analysis, we selected a resolution of 0.2, which represented the point just before the appearance of unstable transitions (indicated by transparent arrows), suggesting stable and biologically meaningful partitions. Notably, subsequent subclustering analyses were performed on specific cell populations of interest to resolve finer cellular heterogeneity where necessary. We also evaluated higher resolutions (e.g., 0.3, 0.5, and 0.8), but observed that they introduced a number of small, unannotatable clusters, suggesting overclustering (Figure S1I). Given our focus on identifying major cell types and ensuring biological interpretability, we selected a conservative resolution of 0.2 at this stage.

### Cell annotation and cellular fraction calculation

We annotated the cell types according to the canonical cell type-specific genes: Epithelial cells (EPCAM, CLDN7, MUC1, CDH1 and KRT20), T/NK cells (CD3E, CD3D, CD8A, CD4, CCR7, IL7R, KLRD1, FGFBP2, GNLY and NKG7), B cells (CD79A, MS4A1 and CD19), Myeloid cells (CD14, CD68, LYZ, CD86, ITGAM, FCGR3B, FCGR3A, CX3CR1, CXCR1, C1QA, C1QB, C1QC and CD163), Plasma cells (MZB1, SDC1 and IGHG1), Fibroblasts (COL1A2, COL3A1, COL14A1, PDGFRB and FGF7), Endothelial cells (CDH5, PECAM1, VWF and DCN), Mast cells (TPSB2 and TPSAB1) and Cycling cells (MKI67 and TOP2A). We also calculated the fractions of each cell type and compared the different distributions between immunotherapy-resistant and sensitive groups.

### Re-clustering and annotation of broad cell types

To further explore the characteristics of various cell types, we used “subset” function to extract different cell subtypes from the initial Seurat object and repeated the analytical process from data normalization to cell type annotation.

### Identification of differential expressed marker genes

Differential expressed marker genes for specific clusters or cell types were identified using “FindAllMarkers” function in Seurat package with a cutoff of min.pct = 0.1 and logfc.threshold = 0.25. Top5 marker genes with highest abstract of log2Foldchange were labeled in heatmaps or volcano plots.

### Signature score calculation at single cell level

We calculated gene signature scores at single cell levels using Gene set variation analysis (GSVA) method by GSVA package (version 1.42.0) [28] and “AddModuleScore” function in Seurat package. To estimate the activity of biological pathways related to inflammation and immune regulation, we chose the gene sets from Hallmark pathways (h.all.v7.2.symbols.gmt) in MSigDB database (https://www.gsea-msigdb.org/gsea/msigdb/). For the calculation of signature scores in different cell types or sample types in scRNA-seq data, the average expression level in each sample type or cell type was counted by “AverageExpression” function. In order to quantify the activity score of inflammatory response pathway across single cells, we applied AUCell algorithm in scRNA-seq data. AUCell scoring process was carried out by extracting single-cell gene expression matrix and calculating an area under the curve (AUC) score for each cell based on the expression ranking of genes in the gene sets. Cells with higher AUCell scores were classified as exhibiting higher pathway activity.

### Definition of signature scores

The cytotoxicity and exhaustion scores for CD8 + T cells were determined based on published signature genes from gene lists of Hu et, al [29]. The signature scores for different phenotype of macrophages were estimated according to LM22.xls from CIBERSORT [30]. We collected signatures of T cell activated pathways based on canonical gene markers (Table S2).

### Cell trajectory analysis

Monocle2 (version 2.22.0) was employed to infer cell differentiation process of CD8 + T cells, monocytes, macrophages and neutrophils [31]. We chose hypervariable genes and used them to order cells in pseudo-time. After dimensionality reduction and cell sorting, the differentiation trajectory was inferred with default parameters. We plotted the cell trajectory classified by different cell states, cell subtypes or sample types. We also demonstrated the dynamic expression level along the pseudo-time for genes we interested in.

### Signature score calculation at bulk RNA-seq level

Top5 marker genes of CEMIP+ Monocytes, CCL4 + Neutrophils and MMP3 + Fibroblasts were selected to form the signature gene sets and GSVA package was applied to perform the calculation (Table S2). Patients were divided into high and low signature groups according to the median value of signature scores.

### Construction and validation of an inflammation-associated classifier model

To predict immunotherapeutic responses, we constructed a classifier model using Random Forest (RF) algorithm and compared it with several other machine learning methods. The modeling procedure included the following steps:

1. Feature selection: To identify representative features of inflammation-related cell subgroups, we applied “FindAllMarkers” function in Seurat package to extract marker genes of the three inflammation-associated cell types. The selection criteria were set as follows: min.pct = 0.1, logfc.threshold = 0.25, and adjusted *P*-value < 0.05. Only genes that met these thresholds in all of the three cell subsets were retained as input features for subsequent model construction. This step ensures that the selected genes exhibit significant differential expression and represent the biological characteristics of the inflammation-associated cell populations, thereby improving the discriminative power of the classifier.
2. Data preprocessing: The expression profile of selected genes was extracted from raw expression matrix of IMvigor 210. Genes with low expression (mean expression ≤ 0) were removed.
3. Clinical label integration: Samples were annotated using response status, ensuring only matched samples across expression and response information were retained. The outcome variable (“Type”) was binary-labeled as “Response” vs. “Non-Response”.
4. Train-test splitting: The dataset was randomly split using stratified sampling by “createDataPartition” function into a training set (70%) and test set (30%), which involved 209 (161 non-responders and 68 responders) and 89 (61 non-responders and 20 responders) samples respectively.
5. Model construction and comparison: Multiple machine learning models were trained using the caret package with 5-fold cross-validation. The models included: Random Forest (RF), Support Vector Machine (SVM), Gradient Boosting (GBM), Logistic regression (Logit) and Extreme Gradient Boosting (XGB). All models were trained using the formula: Type ~ ., where all selected gene expression values served as predictors.
6. Key parameters of RF model: The RF model, which showed competitive performance, was trained with default parameters, and mtry was internally tuned based on accuracy. The RF model builds an ensemble of decision trees using bootstrapped subsets and randomly selects predictors at each split. No manual hyperparameter tuning was required due to the internal resampling-based optimization. Likewise, we did not apply sampling or re-weighting strategies to balance class sizes in order to preserve the natural distribution of the real-world cohort.
7. Model evaluation: The performance of classifier models was assessed using AUC (Area Under the Curve) values in ROC (Receiver Operating Characteristic) curves and confusion matrices. Model explanation was performed by DALEX package and probability-based prediction functions. The prediction probabilities were extracted for each model (type = “prob”) and compared on the test set. To assess the predictive contribution of each input gene, we computed feature importance scores using the “variable_importance” function from DALEX package. Importance scores were calculated for RF model and the top 10 most important genes were visualized.
8. External validation: Two independent cohorts were used for external validation. The RF model trained on IMvigor 210 cohort was applied to the new cohorts. ROC curves were plotted, and AUC values were calculated to confirm the accuracy of the model.

### Subgroup analysis

To investigate the biological interpretability of the model and elucidate potential mechanisms underlying the predicted responders and non-responders, we applied the RF model to TCGA-CRC dataset, stratifying the patients into potential responders and non-responders for subsequent subgroup analyses. We then performed a series of downstream analyses, including immune infiltration estimation, immunotherapeutic response prediction, tumor mutation burden (TMB) assessment, survival analysis, as well as DEGs identification and GSEA analysis.

#### Immune infiltration analysis

CIBERORT is a method to estimate the relative abundance of 22 types of immune cells from bulk RNA-seq data using deconvolution algorithm [30]. We calculated the infiltration level of immune cells by CIBERSORT algorithm and compared the differences of immune cell proportion and expression of common immune checkpoint genes in TCGA-CRC dataset by Wilcox rank sum test.

#### Immunotherapeutic response analysis

TIDE database (http://tide.dfci.harvard.edu/query/) was used to evaluate the potential clinical efficacy of immunotherapy. Higher TIDE scores indicate less benefit from ICI immunotherapy as it indicates a greater probability of immune escape. Exclusion scores are used to assess the ability of tumor cells to resist infiltration by immune cells, reflecting the prevention of immune cells from exerting function in TME. Dysfunction scores focus on the level of impairment of immune cells to exert effector function. Wilcox rank sum test was used to evaluate the differences between different groups. Spearman correlation analysis was applied to investigate the relationship between signature scores and the value of above metrics.

#### TMB and survival analysis

We conducted mutation analysis to investigate whether genetic alterations were associated with the potential responders and non-responders. TMB was defined as the mutation rate per million bases, which was assessed by dividing non-synonymous mutation numbers by the exon length [32]. We examined the difference of TMB between responders and non-responders by Wilcox rank sum test. Kaplan-Meier survival analysis and log-rank test was performed using packages survminer (version 0.4.9) and survival (version 3.2–13) to compare the survival status between the two groups.

#### DEGs identification and GSEA

In order to identify DEGs between potential responders and non-responders, we used DESeq2 package (version 1.34.0) under a cutoff of |log2FoldChange| > 1 and FDR < 0.05. We sorted all genes according to log2FC and performed GSEA using “GSEA” function from package clusterProfiler (version 4.2.2) with a threshold of |NES|>1 and *p* < 0.05 [33, 34]. The gene set “c2.cp.kegg.v7.5.1.symbols.gmt” was downloaded from MSigDB database.

### Statistics

All statistical analyses and visualization were performed using R software (version 4.1.3). The significance of differences between different response groups and cell types was analyzed using Wilcox rank sum test. In all comparisons, a two-tailed *P* value of less than 0.05 was considered statistically significant.

## Results

### Single cell transcriptome atlas of CRC patients received anti-PD1 immunotherapy

**Fig. 1:**
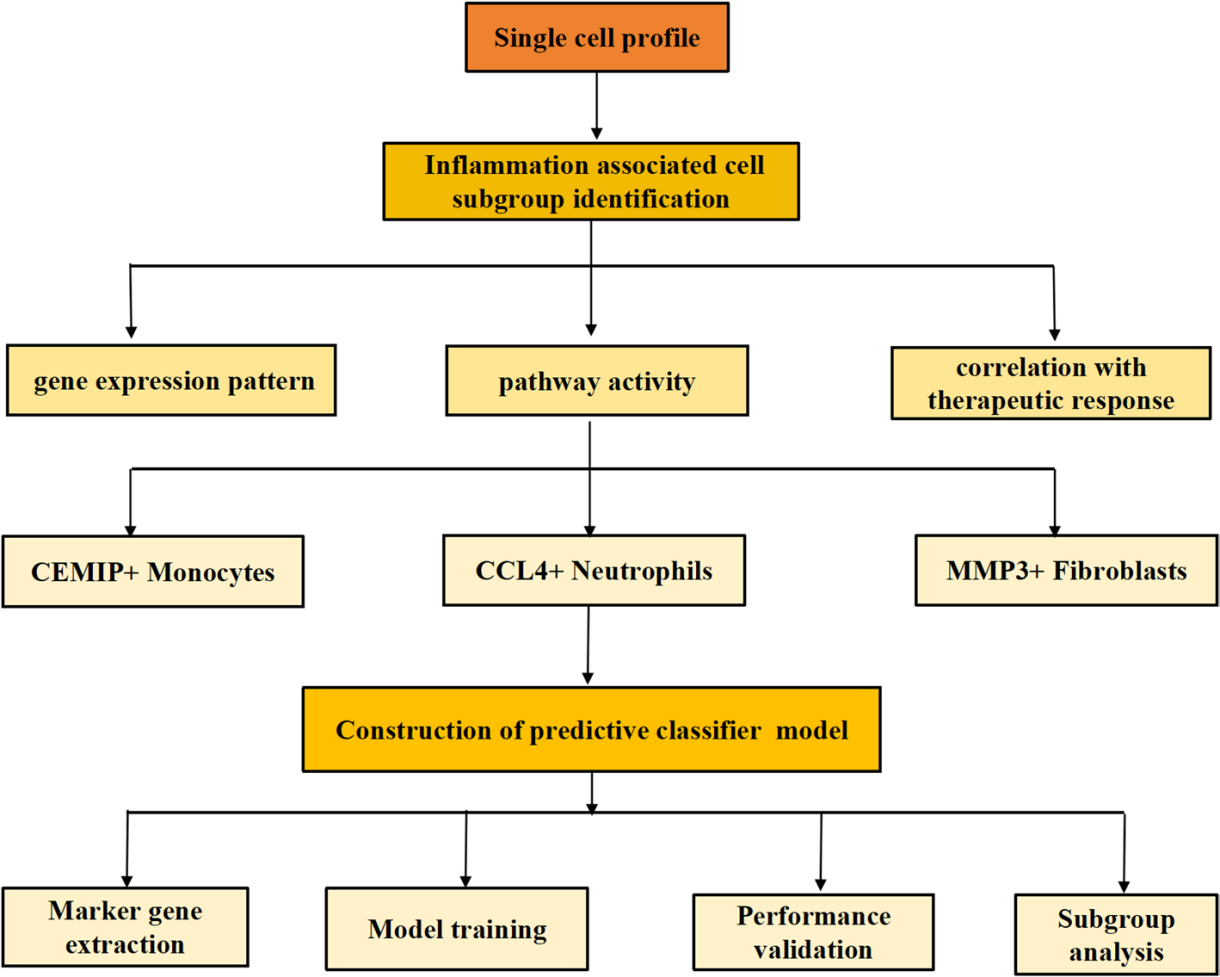
Workflow displaying the research design of present study

**Fig. 2:**
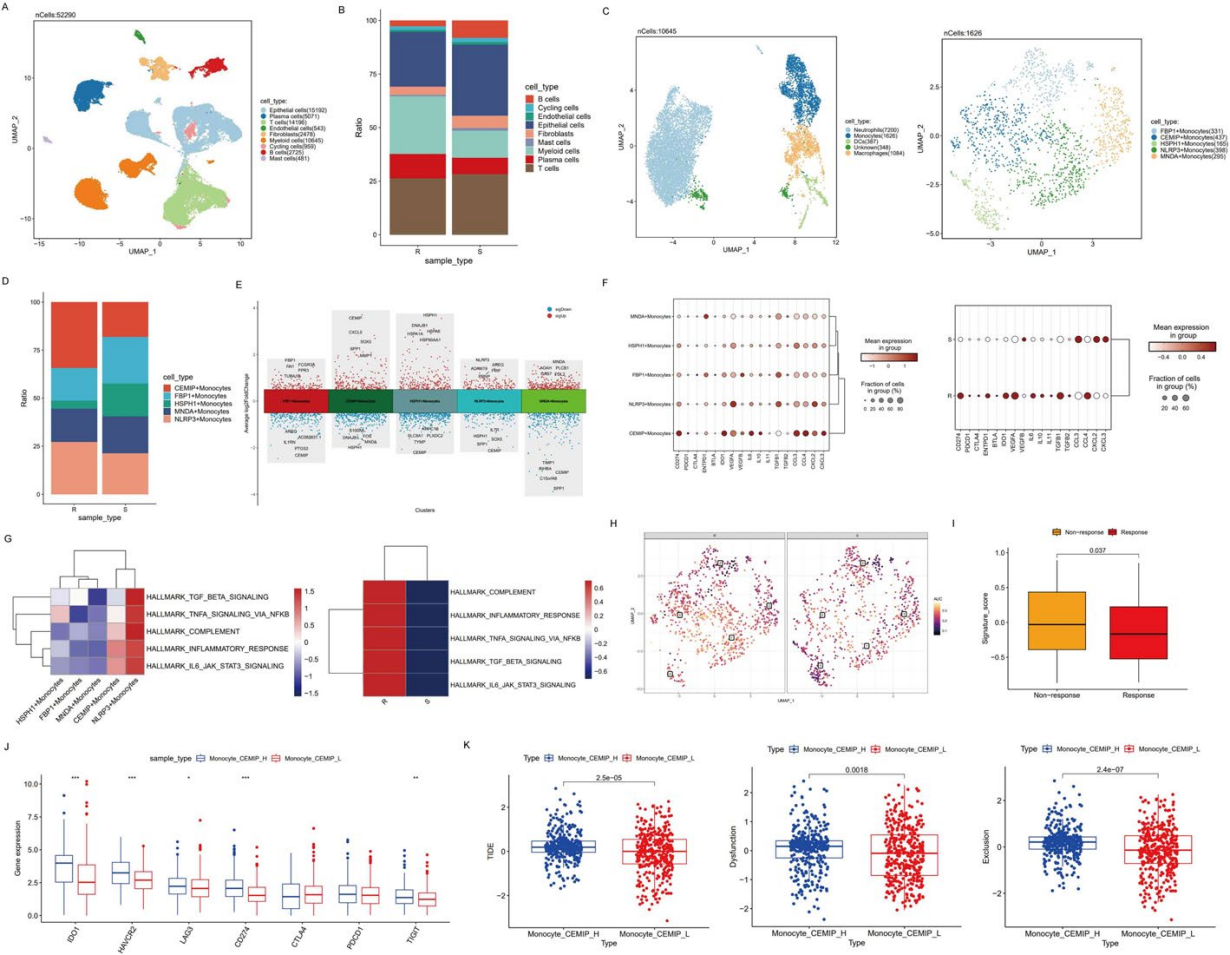
Identification of distinct subsets of myeloid cells in immunotherapeutic resistant group. **A** UMAP plot of all cell types. **B** Bar plot of cell type fractions in total cells for different sample types. **C** UMAP plot of total myeloid cells (left) and monocytes (right). **D** Cellular fraction of each monocyte subtype in immunotherapy-resistant and sensitive group. **E** Volcano plot showing top marker genes of monocytes subtypes. **F** Dot plot showing the expression of pro-inflammatory and immunosuppressive factors in different monocyte subtypes (left) and sample types (right). **G** Heatmap demonstrating enrichment scores of inflammatory response and immune suppressive associated pathways in different monocytes subsets (left) or sample types (right). **H** AUCell score for the pathway activity of inflammatory response in different sample types. **I** Boxplot of signature scores for CEMIP+ Monocytes in responders and non-responders in IMvgior210 cohort. **J** Differences of expression levels of immune checkpoint genes in CEMIP+ Monocytes signature score high and low groups in TCGA-CRC dataset. **K** The differences of TIDE scores, Dysfunction scores and Exclusion scores between CEMIP+ Monocytes signature score high and low groups in TCGA-CRC dataset

The schematic diagram of this study was presented in Fig. 1. The results of pre-processing and quality control for scRNAseq data were illustrated in Table S1and Fig S1A-D. After the filtering of low-quality cells, 52,290 cells and 31,107 genes from 6 patients after PD1 blockade immunotherapy were obtained for single cell transcriptome analysis. Three patients were divided into immunotherapy-resistant group, who showed a progressed disease state evaluated by radiological examination. The other three who captured partial relief after the treatment were categorized into immunotherapy-sensitive group. All the cells were clustered into 15 groups through data integration, unsupervised clustering and UMAP dimensionality reduction (Fig S1H), with no apparent batch effects between different samples and obvious impact caused by cell cycle confounders (Fig S1E-F). UMAP visualization of mitochondrial gene expression revealed that high expression levels of mitochondrial gene were predominantly observed in epithelial cells, while immune and stromal cells exhibited uniformly low mitochondrial gene expression (Figure S1F). Initial clustering was performed with a resolution of 0.2, as determined via the clustree package (Figure S1G). This resolution yielded stable cluster structures and allowed for clear annotation of major cell types based on canonical marker gene expression (Figure S1J-K).Although a higher number of clusters could be obtained at resolutions ≥ 0.3, these resulted in overclustering, including the emergence of unassignable small cell populations with no clear marker expression (Figure S1I). Depending on the expression level of well-known marker genes in each cluster, 9 cell types including epithelial cells, T cells, B cells, myeloid cells, plasma cells, fibroblasts, endothelial cells, mast cells and cycling cells were identified (Fig. 2A and Figure S1J-K). Then, we calculated the fraction of each cell types in different groups. The percentage of myeloid cells and epithelial cells varied to an extent between the two groups. Patients resistant to immunotherapy showed a lower ratio of epithelial cells and a rising trend of myeloid cell proportion (Fig. 2B).

### The landscape of exhausted T cells in immunotherapy-resistant TME

Although the distribution of T cells did not differ apparently between two groups, they were enriched in TME and exerted important roles in immune regulation. In order to characterize the heterogeneity of T cells, we re-clustered initial T cells into CD4 + T cells, CD8 + T cells, Regulatory T cells (Tregs, a special subtype of CD4 + T cells) and NK cells based on conventional markers (Fig S2A and S2B). We observed an increased proportion of CD4 + T cells, Tregs and decreased CD8 + T cells and NK cells in immunotherapy-resistant group (Fig S2C). The expression levels of canonical marker genes for T/NK cells subtypes were illustrated in Fig S2D. Since MSI-H tumors were reported to have a higher infiltration proportion of CD8 + T cells in TME, we then focused on CD8 + T cells. First, CD8 + T cells were re-clustered into seven subclusters and defined by the top differential expressed marker genes (Fig S2E-F), including two types of Naive CD8 + T cells (GZMK+CD8 + T and LTB+CD8 + T), two types of Resident CD8 + T cells (KLRC2 + CD8+T and IL7R+CD8 + T), a subgroup of Cytotoxic CD8 + T cells (FGFBP2 + CD8+T) and two types of Exhausted T cells (GZMB+CD8 + T and CXCL13 + CD8+T). Exhausted CD8 + T cells expressed not only GZMA, GZMB, GZMK, PRF1, IFNG, GNLY and NKG7, which indicated for cytotoxic effector functions, but also markers of coinhibitory receptors (PDCD1, HAVCR2, LAG3 and TIGIT) (Figure S2H). Compared to immunotherapy-sensitive group, resistant group presented a higher infiltration of Exhausted and Naive CD8 + T cells (Fig S2G and S2I). Cell trajectory analysis further validated the differentiation trajectories of the above CD8 + T cell subgroups, which initialed from Naive T cells and ended up with Exhausted T cells and Resident T cells (Fig S2J). We then calculated the cytotoxic and exhausted signature scores for CD8 + T cells in different cell types and sample types. Results showed that Exhausted CD8 + T cells exhibited high levels of both cytotoxic signature score and exhausted score. Compared to immunotherapy-resistant group, sensitive group illustrated both higher cytotoxic score and exhausted score (Fig S2K). In order to evaluate the exhausted level in TME, we compared the expression of immune checkpoint genes and T cell-activated related genes between the two groups. The results showed that expression levels of activated T cell-related genes were higher in immunotherapy-sensitive group and resistant group exhibited increasing expression of immune checkpoint genes (Fig S2M). We also calculated and compared pathway activities of several T cell-activated and inhibited pathways using “AddModuleScore” method based on gene sets composed of canonical genes. Among them, pathways associated with T cell-activating including IFNG, co-stimulatory, IL2, and NKκB signaling were activated in immunotherapy sensitive group, while pathways related to negative regulation of T cells, including TGFB and immune checkpoint signaling were enriched in resistant group (Fig S2L). In addition to CD8 + T cells, Tregs in immunotherapy-resistant group also expressed high level of immune checkpoint genes, indicating an exhausted immune microenvironment that probably leading to the resistance to immunotherapy (Fig S2N). In summary, we revealed an exhausted profile of T cells in the TME of immunotherapy-resistant patients.

### The remodeling of myeloid cells identified a subpopulation of monocytes associated with unfavorable responses to immunotherapy

Next, we analyzed the remodeling of myeloid cells. We re-clustered and annotated the myeloid cells into monocytes, macrophages, dendritic cells and neutrophils determined by the expression of specific markers (Fig. 2C and S3A-B). Fig S3C presented a higher proportion of neutrophils in immunotherapy resistant group. Myeloid cells have been reported to execute complicated functions in regulating TME, which could simultaneously function as anti-tumor effectors or promote tumor progression. We first focused on monocytes, re-clustered the population into 5 subclusters and defined them by top marker genes, including FBP1 + Monocytes, CEMIP+ Monocytes, HSPH1 + Monocytes, NLRP3 + Monocytes and MND1 + Monocytes (Fig. 2C and E). Through the comparison for the distribution of these subtypes between immunotherapy-resistant and sensitive groups, we noticed that the proportion of CEMIP+ Monocytes was increased in resistant group (Fig. 2D). Gene expression analysis revealed that CEMIP+ Monocytes expressed the highest level of proinflammatory factors (IL6, IL10, CCL2, CCL3, CCL4, CXCL2, CXCL3), vascular endothelial growth factor (VEGFA and VEGFB), as well as immune inhibitory genes (CD274, PDCD1, IDO1), suggesting the immunosuppressive function of CEMIP+ Monocytes. Most of these genes also showed an increasing expression level in immunotherapy-resistant group than sensitive group (Fig. 2F). To characterize the inflammatory and immunosuppressive features in TME, we selected relevant pathways from HALLMARK gene set and performed GSVA analysis to calculate pathway activities. We found that CEMIP+ Monocytes and NLRP3 + Monocytes, which were both enriched in immunotherapy resistant group, showed the highest enrichment scores of most inflammation-related and immune suppressive pathways. Compared to immunotherapy sensitive group, inflammation-related pathways in therapeutic resistant samples were activated, especially in CEMIP+ Monocytes and NLRP3 + Monocytes subsets (Fig. 2G-H). In order to determine whether the signature of CEMIP+ Monocytes could be applied as a negative biomarker to predict immunotherapy responses, we conducted GSVA analysis to calculate the signature score of CEMIP+ Monocytes for each sample in IMvigor210 cohort and TCGA-CRC dataset. We discovered that high signature group had significantly higher expression levels of immune checkpoint genes (IDO1, HAVC2, LAG3, CD274 and TIGIT) in TCGA-CRC cohort (Fig. 2J), and patients poorly responded to immunotherapy tended to have significantly higher CEMIP+ Monocytes signature scores (Fig. 2I). Immunotherapeutic response analysis also revealed that high signature group had significantly higher TIDE scores, Dysfunction scores and Exclusion scores than low signature group (Fig. 2K), indicating that TME prevented immune cells infiltrating and immune cells themselves were dysfunctional, leading to a higher probability of immune escape and treatment failure. These results suggested that CEMIP+ Monocytes could serve as an effective predictor for ICI therapy responses.

We also investigated the macrophages. After re-clustering, four subclusters of macrophages were defined by top marker genes (Fig S2D-F). IGHA1 + Macrophages had a higher proportion in immunotherapy-resistant group (Fig S2G). We evaluated the signature scores of M1, and M2 phenotypes. The results showed that IGHA1 + Macrophage exerted highest M2 signature, which suggested a pro-tumor environment. However, when comparing signature scores between immunotherapy-resistant and sensitive groups, the resistant group exhibited an increased M1 signature and decreased M2 signature (Fig S2H). Cell trajectory analysis illustrated that the two types of inflammation associated monocytes (CEMIP+ Monocytes and NLRP3 + Monocytes) were at the initial status of the differentiation trajectories and IGHA1 + Macrophages were at the terminal status (Fig S2I).

### A subset of aged neutrophils modulated pro-tumor inflammation and was associated with suppressive immune microenvironment

**Fig. 3:**
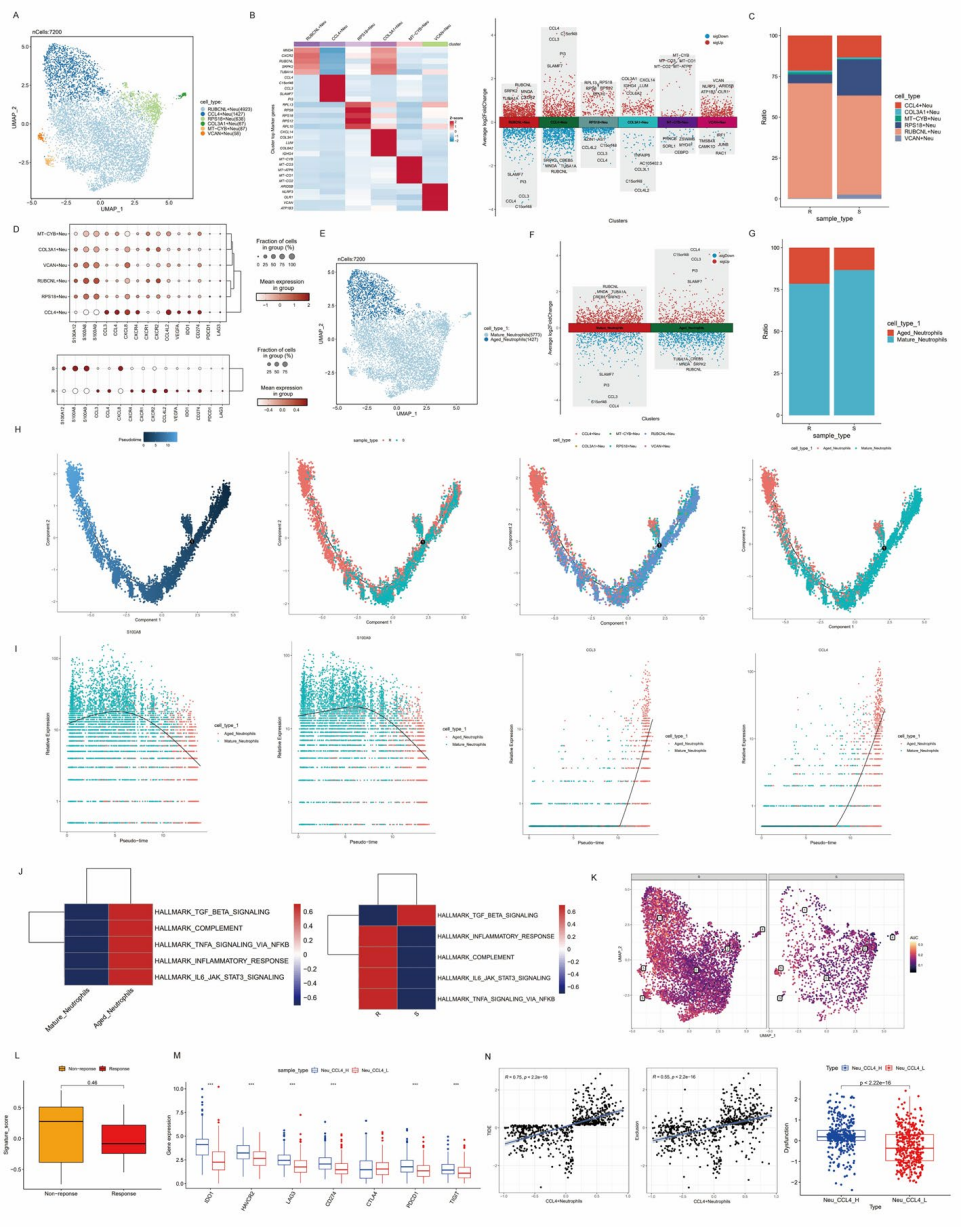
Remodeling of neutrophils in immunotherapy-resistant group. **A** UMAP plot of neutrophils. **B** Heatmap (left) and volcano plot (right) displaying cluster-specific genes. **C** Bar plot of cell type fractions in different sample types. **D** Dot plot of inflammatory response and immune suppressive genes in different neutrophil subtypes (up) and sample types (down). **E** UMAP plot of two defined neutrophil subsets. **F** Volcano plot showing marker genes of neutrophil subsets. **G** Bar plot of cell type fractions in different sample types. **H** Pseudo-time trajectory showing the differentiation process of neutrophils. **I** The dynamic expression level of CCL3, CCL4, S100A8 and S100A9 along the pseudo-time. **J** GSVA enrichment scores for inflammatory response and immune suppressive pathways in different subsets of neutrophils and in different sample types. **K** AUCell score for the pathway activity of inflammatory response in different sample types. **L** Boxplot of signature scores for CCL4 + Neutrophils in responders and non-responders in GSE78220 cohort. **M** Differences of expression levels of immune checkpoint genes in CCL4 + Neutrophils signature score high and low groups in TCGA-CRC dataset. **N** Correlation between CCL4 + Neutrophils signature scores and TIDE (left) or Exclusion (middle) score and boxplot of Dysfunction score for CCL4 + Neutrophils signature score groups (right) in TCGA-CRC dataset

As mentioned above, the percentage of neutrophils was increased in immunotherapy resistant patients. We re-clustered neutrophils into six subclusters and defined each subcluster by top marker genes, including RUBCNL+ Neutrophils, CCL4 + Neutrophils, RPS18 + Neutrophils, COL3A1 + Neutrophils, MT-CYB+ Neutrophils and VCAN+ Neutrophils (Fig. 3A-C). According to the expression patterns of different neutrophil clusters, we further divided them into two distinct subgroups, including a mature-neutrophil subtype, distinguished by the higher expression of granule-related genes (S100A8 and S100A9) and an aged- neutrophil subtype highly expressing CCL4 and other chemokines (Fig. 3D-F). To explore the reprogramming of neutrophils in patients resistant to immunotherapy, we compared the infiltration of the two types neutrophils in two groups and noticed that the fraction of aged neutrophils was increased in resistant group (Fig. 3G). Trajectory analysis confirmed that the mature subset was the root and the aged subset was at the end-point of the pseudo-time (Fig. 3H). The expression of S100A8 and S100A9 declined while CCL3 and CCL4 rosed along this trajectory (Fig. 3I). We also explored the inflammation-related pathways from HALLMARK gene set using GSVA and AUCell analysis. Compared to mature neutrophils, aged neutrophils were enriched in inflammation-related and immune suppressive pathways and the enrichment scores of these pathways were higher in therapeutic resistant group (Fig. 3J-K), suggesting that the mechanism contributing to the resistance to immunotherapy might be the failure of inflammation resolution mediated by the senescence of neutrophils. To further evaluate the immunosuppressive function of CCL4 + Neutrophils, we used the top5 marker genes to calculate the CCL4 + Neutrophil signature score of samples in bulk RNA-seq datasets by GSVA analysis and classified patients into high signature score group and low signature score group. In GSE78220 cohort, patients poorly responded to immunotherapy also tended to have higher CCL4 + Neutrophils signature scores, though the difference was not statistically significant (Fig. 3L). Gene expression analysis in TCGA-CRC dataset demonstrated that higher score of CCL4 + Neutrophil signature was significantly associated with increasing expression of immune checkpoint genes (CTLA4, PDCD1, CD274, HAVCR2, LAG3 and IDO1), reflecting an immunosuppressive phenotype (Fig. 3M). We also applied TIDE algorithm to TCGA-CRC dataset and observed a significantly positive correlation between CCL4 + Neutrophils signature score and TIDE or Exclusion score. Boxplot illustrated that high signature group had significantly higher Dysfunction scores (Fig. 3N). These results suggested that CCL4 + Neutrophil could serve as an effective predictor for ICI therapy responses.

### Fibroblast sub-clustering revealed distinct populations associated with inflammatory response and the potential to predict therapeutic outcome

**Fig. 4:**
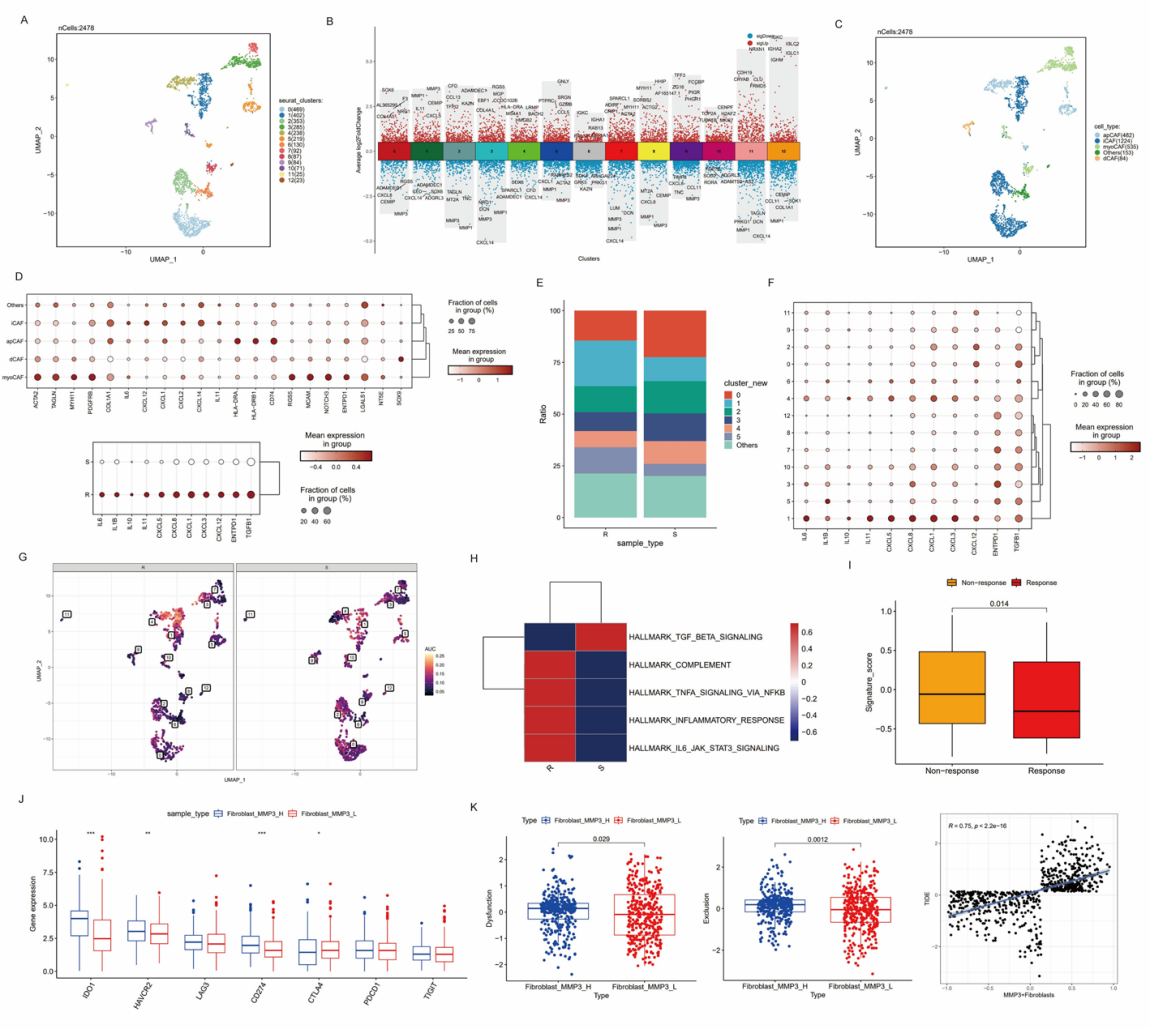
Fibroblasts heterogeneity analysis. **A** UMAP plot of fibroblasts. **B** Volcano plot of cluster-specific genes. **C** UMAP plot of defined fibroblasts subgroups. **D** Dot plot demonstrating the expression of canonical genes in defined fibroblasts subsets (up) and the expression of pro-inflammatory factors in different sample types (down). **E** Bar plot of cluster fractions in immunotherapy-resistant and sensitive group. **F** Dot plot showing pro-inflammatory genes expression in fibroblasts subtypes. **G** AUCell score for the pathway activity of inflammatory response in different sample types. **H** GSVA enrichment scores for inflammatory response and immune suppressive pathways in different sample types. **I** Boxplot of signature scores for MMP3 + Fibroblasts in responders and non-responders in IMvigor210 dataset. **J** Differences of expression levels of immune checkpoint genes in MMP3 + Fibroblasts signature score high and low groups in TCGA-CRC dataset. **K** Correlation between CCL4 + Neutrophils signature scores and TIDE, Dysfunction, Exclusion scores inferred from TIDE algorithm

We continued to explore the reprograming of fibroblasts. Fibroblasts were re-clustered into 13 clusters and top marker genes were identified (Fig. 4A-B). Fibroblasts from cluster 0, 1, 2 showed high expression of chemokines (IL6, CXCL12, CXCL1, CXCL14 and IL11), similar to the previously reported inflammatory CAFs (iCAFs) [35]. Cluster 3, 7, 8,10 showed high expression levels of several ECM remodeling genes (ACTA2, COL1A1, TAGLN, MYH11, PDGFRB), which were reported as matrix CAFs (mCAFs) [36]. Cluster 4, 5, 11 expressed high level of antigen presentation genes and was defined as antigen presentation CAFs (apCAF). Cluster 9 was defined as differential CAFs (dCAFs) with high expressing of stem cell differentiation-related gene SOX9 (Fig. 4C-D). The proportions of cluster 1 and cluster 5 were higher in immunotherapy-resistant group than sensitive group (Fig. 4E). Most pro-inflammatory and immune suppressive factors (IL6, IL10, IL11, IL1B, CXCL5, CXCL8, CXCL1, CXCXL3, CXCL12, ENTPD1 and TGFB1) were upregulated in immunotherapy-resistant group (Fig. 4D and F). We also explored pathway activities from HALLMARK gene set using GSVA and AUCell analysis. The results showed that most inflammation-related and immune suppressive pathways were enriched in immunotherapy-resistant group and the AUCell score of inflammatory response pathway in cluster 1 (MMP3 + Fibroblasts) was higher in therapeutic resistant group (Fig. 4G-H). We then wondered whether the signature of MMP3 + Fibroblasts could serve to indicate poorer anti-tumor immunity and unfavorable responses to immunotherapy. The top5 marker genes was used to calculate MMP3 + Fibroblasts signature scores in IMvigor210 dataset and patients were divided into high and low signature score groups. We discovered that the signature scores of MMP3 + Fibroblasts were significantly higher in non-responders than responders (Fig. 4I). We also observed a significantly upregulated expression of immune checkpoint genes (IDO1, HAVCR2 and CD274) in high signature group in TCGA-CRC dataset (Fig. 4J). The Dysfunction and Exclusion scores inferred from TIDE algorithm were significantly higher in high signature group and TIDE scores showed a significantly strong positive correlation with MMP3 + Fibroblasts signature scores (Fig. 4K). In summary, these results highlighted on the tumor-promoting inflammation of MMP3 + Fibroblast subsets, which might work together with pro-inflammatory myeloid cells to form an immunosuppressive TME.

### Construction and validation of an inflammatory signature-associated classifier model derived from monocytes, neutrophils and fibroblasts

**Fig. 5:**
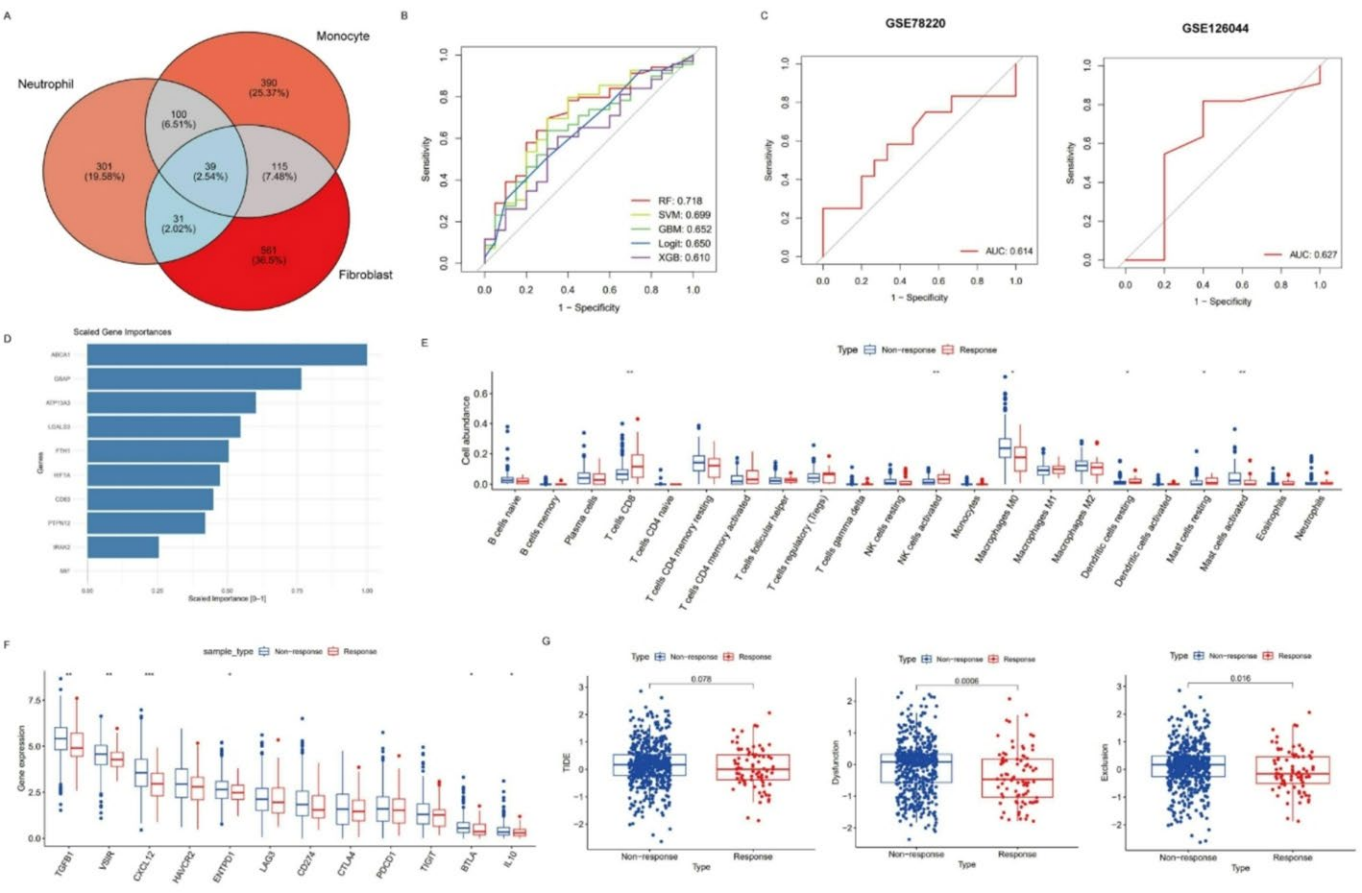
The establishment and validation of the inflammation-related classifier model. **A** Venn plot of the shared marker genes identified in all inflammation-associated cell subgroups. **B** ROC curves showing the efficacy of models generated by multiple machine learning methods. **C** ROC curves verifying the robustness and accuracy of the model in other two external validate cohorts. **D** Bar plot of the top10 important genes in the RF model. **E** The differences of immune cell proportion between potential responders and non-responders generated by the classifier model. **F** Differences of immune suppressive genes expression between potential response and non-response group produced by the classifier model. **G** Boxplot reveals the correlation between the predicted classification results and immune escape score inferred from TIDE algorithm

Considering the potential role of inflammation-associated cell subpopulations in modulating TME and affecting immunotherapeutic responses, we developed a classifier model by machine learning algorithm to predict immunotherapeutic responses based on the features of inflammation-related cell subgroups. To screen out essential key genes characterizing the features of inflammation-related cell subgroups, we identified the significantly differential expressed marker genes of CEMIP+ Monocytes, CCL4 + Neutrophils and MMP3 + Fibroblasts and extracted 39 intersect genes to train the classifier model (Fig. 5A). Five machine learning methods were employed, including RF, SVM, GBM, Logit and XGB. We split the IMvigor210 cohort. 70% number of the cohort was set as a training dataset and the remaining 30% as an internal validate dataset. The model’s performance was assessed using ROC curves. All of the algorithms demonstrated satisfying performance with AUCs over 0.6 and the best-performing model was RF, achieving the highest AUC of 0.718, so we selected the RF model as the optimal classifier model to predict therapeutic responses (Fig. 5B). During the process of model training, hyperparameter tuning was performed using 5-fold cross-validation. The final RF model employed the optimal value of mtry = 2, which yielded the highest mean accuracy of 0.756 across the validation folds. The top10 genes with highest importance scores in RF model were illustrated in Fig. 5D. The model’s robustness and accuracy were further validated using two independent external validation datasets (AUC > 0.6), indicating the potential for effective patient stratification (Fig. 5C).

### Differential immune microenvironment, clinical characteristics and signaling pathways in potential responders and non-responders generated by classifier model

To further elucidate the biological relevance of our model, we applied it to a larger, independent TCGA cohort and compared key biological differences between the produced responder and non-responder subgroups. First, 84 responders and 617 non-responders in TCGA-CRC dataset were generated by the classifier model. After removing samples from normal tissues and those without available clinical information, a total of 80 responders and 536 non-responders were included in the subsequent analyses. We then explored the biological characteristics distinguishing the predicted responders and non-responders. Immune infiltration analysis revealed differences in immune microenvironment, while immunotherapeutic response prediction suggested varying likelihoods of benefit from immune checkpoint blockade, allowing cross-comparison with our model-predicted responses for mutual corroboration. Assessment of TMB and survival highlighted genomic and prognostic disparities. DEGs identification and GSEA analysis uncovered pathways potentially mediating therapeutic resistance. We employed CIBERSORT algorithm to estimate the infiltration levels of immune cells. As was demonstrated in Fig. 5E, the abundance of CD8 + T cells and activated NK cells was significantly upregulated in responders, suggesting a stronger anti-tumor immunity. The expression levels of conventional immune checkpoint genes were upregulated in non-responders, although did not show statistical significance. Other genes that negatively regulated anti-tumor immunity (TGFB1, VSIR, CXCL12, ENTPD1, BTLA and IL10) exhibited significantly higher expression level in non-responders (Fig. 5F). Notably, non-responders had significantly higher Dysfunctional and Exclusion scores than responders. The non-response group also showed an increasing trend of TIDE score (Fig. 5G). This suggested that patients in response group were more likely to benefit from immunotherapy, which was consistent with the expected results predicted by our classifier model. TMB is also considered as a biomarker for immunotherapeutic response and patients with high TMB benefited more from immunotherapy. As shown in Fig S4B, TMB showed increased trend in potential responders, but did not differ significantly. Then, we combined the clinical and survival information with response status to figure out the correlation between therapeutic response and clinicopathological features. Fig S4A demonstrated that potential non-responders tended to have a more advanced and progressive disease status. The fraction of patients in Stage IV, T4 stage, N2 stage and M1 stage were higher in non-responders than responders. The overall survival (OS) of non-responders was also worse than responders, but the difference was not statistically significant (Fig S4C). Finally, DEGs between potential responders and non-responders were also identified (Fig S4D). We employed GSEA analysis to figure out the activated pathways in responders (Fig S4E).

## Discussion

Clarifying the mechanisms of therapeutic resistance, predicting the efficacy of immunotherapy, and guiding precision clinical stratification are the essential keys to improve the prognosis of patients with malignant tumors. Although many studies at large transcriptome or genome scale have been conducted to resolve the mechanisms of immunotherapeutic resistance, a transcriptome profiling at single-cell level allows for more precise resolution to discover the unique molecular features and heterogeneity of each cell type. In recent years, many studies combined bulk RNA-seq datasets with scRNA-seq data to apply cell type characterization inferred from scRNA-seq to large clinical cohorts for prognostic model construction. However, a classifier model characterized by inflammation-associated cell signatures aimed to predict therapeutic responses has not been reported yet.

Several studies analyzing scRNA-seq data of MSI-H CRC patients received immunotherapy have been recently reported, one focused on the remodeling of immune and stromal cells, and the comparisons were conducted among treatment-naive, pCR (pathological CR) and non-pCR patients [21]. Another was the dataset we analyzed in present study, which only conducted genes differentially expressed analysis in immunotherapy-resistant patients and selected specific biomarkers for experimental validation [23]. In this study, we analyzed the data in depth to demonstrate the single cell transcriptome profile of patients with different clinical responses and analyzed the entire TME component to investigate the relationship between TME remodeling and tumor responses to immunotherapy, highlighting the distinct characteristics of inflammation-associated cell subsets.

After identifying various cell types in TME, we investigated the immune and stromal components of TME. As is known to all, myeloid cells exert complicated functions in TME [37, 38]. The accumulation of tumor associated macrophages (TAM) and myeloid-derived suppressor cells (MDSCs) in TME is associated with tumor metastasis and resistant to treatment [39, 40]. Therefore, overcoming the immunoinhibitory effects of suppressive myeloid cells is a major challenge in immunotherapy [41]. However, up to date, only a few of therapeutic strategies targeting myeloid cells has been applied to the clinical treatment of cancers due to the insufficient understanding of the mechanisms by which myeloid cells promote tumor development and immune escape [42]. In present study, we found that immunotherapy-resistant group exhibited higher expression of pro-inflammatory factors. Therefore, we proposed the assumption that the inflammatory factors could promote the transformation from anti-tumor to immune-suppressive microenvironment. In monocytes subset, we identified a subgroup of specific CEMIP+ Monocytes with immunosuppressive function and could serve as a potential predictor for immunotherapeutic responses. Previous studies have found that inflammatory response factors in monocytes and macrophages of CRC were significantly upregulated, higher than those of other cell subgroups [43]. CD44 + monocytes had a higher information flow intensity than CD44- monocytes and CD44 had good predictive ability for immune checkpoint blockade responses [44]. Another study discovered a subset of neutrophil-like monocytes increasing in colon cancer patients and was able to induce dysfunctional TIGIT + NK Cells [45].

When it comes to neutrophils, a subset of aged CCL4 + Neutrophil attracted our attention, which were distinguished by the high expression levels of multiple chemokines. Bulk RNA-seq analysis found that higher scores of CCL4 + Neutrophils signature was associate with higher expression of immune checkpoint genes, reflecting an immunoinhibitory function. Interestingly, another research in non-small cell lung cancer also discovered a similar aged neutrophil subset enriched in patients with poorer prognosis to combination immunotherapy and chemotherapy, suggesting a similar mechanism across cancer types [46].A recent research carried out a comprehensive analysis of the landscape of neutrophils heterogeneity in CRC, which defined four subtypes of tumor associated neutrophils (TANs) with diverse functional states and identified specific neutrophil-derived gene signatures associated with poor prognosis [47]. The research about a CRC patient with a local inflammatory condition demonstrated that inflammation mediated by neutrophils promoted resistance to immune checkpoint inhibitors in MSI-H CRC [48]. Compared to the previous classification of TANs which divided TANs into pro-inflammatory subtype and immunosuppressive subtype [49], our study revealed an aged subset of neutrophils enriched in immunotherapy-resistant samples, exhibiting both pro-inflammatory and immunosuppressive functions.

As for the analysis of stromal cells, we also identified a subset of fibroblasts associated with pro-inflammatory and immunosuppressive factors, which were enriched in immunotherapy-resistant group. It has been proved that cancer-associated fibroblasts are heterogeneous cells with different functions [50]. Besides the maintenance and reconstructing of extracellular matrix, they are important in shaping TME by promoting inflammation and cancer development [51, 52]. In several types of cancers, iCAFs were characterized by high expression of cytokines and chemokines, which regulated immune responses and promoted tumor progression [53, 54].

The potential association between inflammation and immunotherapy-induced responses in CRC patients has been confirmed by previous research. A large cohort of scRNA-seq analysis revealed the remodeling of immune and stromal cells by ICI in pCR response has overlap with pro-tumor inflammatory resolution and proinflammatory IL1B+ Monocytes were enriched in non-pCR patients. In addition, proinflammatory factors (IL1A, IL1B, CCL2/3/4, IL6, IL32, CXCL1, CXCL8) from myeloid cells, endothelial cells, and fibroblasts were significantly more highly expressed in the non-pCR group [21].

We introduced a novel classifier model using a combination of scRNA-seq and bulk transcriptomic data, which enabled us to capture both the details of distinct cell populations and broader gene expression landscape. By testing different machine learning algorithms, we identified a RF model that most accurately predicted the therapeutic responses. RF algorithm is an integrated learning method commonly used for classification and regression tasks. It works to make predictions by constructing multiple decision trees. We verified the performance of the model in external datasets and to enhance the biological interpretation of the model, we applied it to an independent, larger TCGA cohort and examined biologically meaningful differences between potential responders and non-responders generated by our classifier model. These analyses revealed encouraging associations between the model-predicted subgroups and various immune-related as well as clinicopathological characteristics. Nevertheless, we acknowledged that the AUC values obtained from external validations indicated only moderate discriminatory ability, which might limit direct clinical translation at this stage. The possibility of overfitting could not be entirely excluded due to the complexity of biological features and the limited sample size. The limited predictive performance may also, in part, be attributable to our decision not to implement specific methods for addressing class imbalance, in order to maintain the integrity of the original dataset. These highlight the need for further refinement to improve the robustness and generalizability of the model. In the future, we plan to explore strategies such as oversampling, undersampling or class weight adjustment to further optimize the model performance. We also aim to validate the model in larger and independent cohorts to enhance its clinical applicability.

Present study has other several limitations. Firstly, the sample size of the scRNA-seq dataset we involved was too small, so there was a risk that the heterogeneity between patients may be more pronounced than actual biological effects. A larger number of CRC samples from multiple centers are necessary to be included. Building upon the insights gained from present study and inspired by the need for cross-cohort robustness, we are actively extending this analysis to multiple immunotherapy cohorts. Secondly, present study just preliminarily explored the cellular features and molecular characteristics that possibly contribute to the resistance to immunotherapy. The potential interactions between tumor cells and TME have not been clearly elucidated. In addition, we recognize the broader potential of incorporating additional hypothesis-driven frameworks, such as modeling cell-cell communication, functional reprogramming and response-specific pseudotime lineage tracing. Inspired by these possibilities, we plan to extend our work in the future by integrating multiple immunotherapy cohorts and leveraging advanced computational tools to further dissect the complex cellular ecosystems underlying therapeutic responses. Another imitation is that our current study is only a correlation analysis based on multidimensional data, which lacks biological experimental validation. Further analysis and experimental validations are needed to confirm the mechanisms of therapeutic resistance and the efficacy of the classifier model also needs to be further verified.

## Conclusion

Our study provided a comprehensive single cell analysis on the remodeling of TME after PD1 blockade immunotherapy. Through analyzing the characteristics of essential cell components in TME, we identified distinct cell types that might serve as biomarkers for therapeutic responses. Our study highlighted a correlation between pro-inflammatory factors and inhibited immune responses. Using random forest algorithm, we constructed an inflammation-related classifier model for the prediction of immunotherapeutic responses. Overall, our study provided a theoretical basis and developed a novel classier model for the precise management of ICI immunotherapy.

## Electronic Supplementary Material

Below is the link to the electronic supplementary material.

Supplementary Material 1.

## Data Availability

The public datasets analyzed in the current study are available in online repositories, including ENA (https://www.ebi.ac.uk/ena/), TCGA database (http://portal.gdc.cancer.gov/projects) and GEO database (https://www.ncbi.nlm.nih.gov/geo/).
