# Supplementary material for: Prediction of immunotherapeutic responses by a classifier model based on inflammation-associated tumor microenvironment signatures in colorectal cancer
Source: Discov Oncol. 2026 Feb 1;17:378. doi: 10.1007/s12672-026-04548-6 (PMC12953840; doi:10.1007/s12672-026-04548-6)
Supplement: Supplementary file 1 — Supplementary Material 1. [file 12672_2026_4548_MOESM1_ESM.docx]

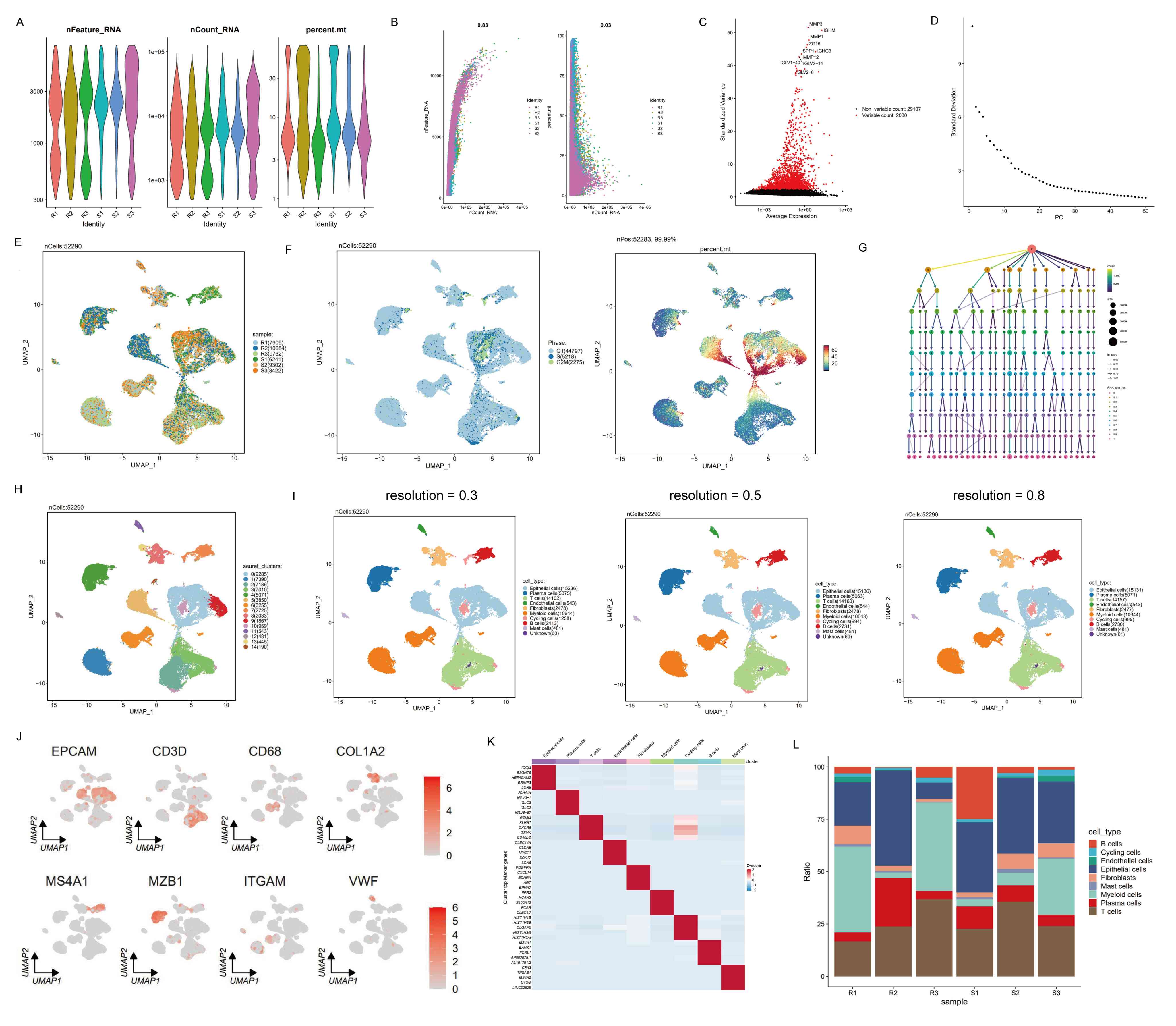


**Figure S1.** The characteristics of scRNA-seq data. (A) Scatter plots displaying the total number of genes (nFeature_RNA), reads (nCount_RNA) and percentages of mitochondrial genes (percent.mt) in unfiltered cells. (B) The correlation between nCount_RNA and nFeature_RNA (left) and between nCount_RNA and percent.mt in unfiltered cells. (C) Top 2000 hypervariable genes were selected for further analysis. (D) Elbow plot showing the relationship between the number of PC dimensions and the standard deviation explained. (E) The UMAP plot of 6 mixed samples. (F) The UMAP plot of cells in different cell-cycle phases (left) and feature plot illustrating mitochondrial gene expression levels (right). (G) clustree plot showing the optimal resolution for cell clustering. (H) UMAP plot of 15 clusters. (I) UMAP plots showing cell annotation results under different clustering resolutions. (J) Feature plots presenting classical marker genes for annotated cell types. (K) Heatmap identifying the expression of top marker genes sorted by average log2FoldChange in annotated cell types. (L) Bar plot of cell type fractions in total cells for different samples.


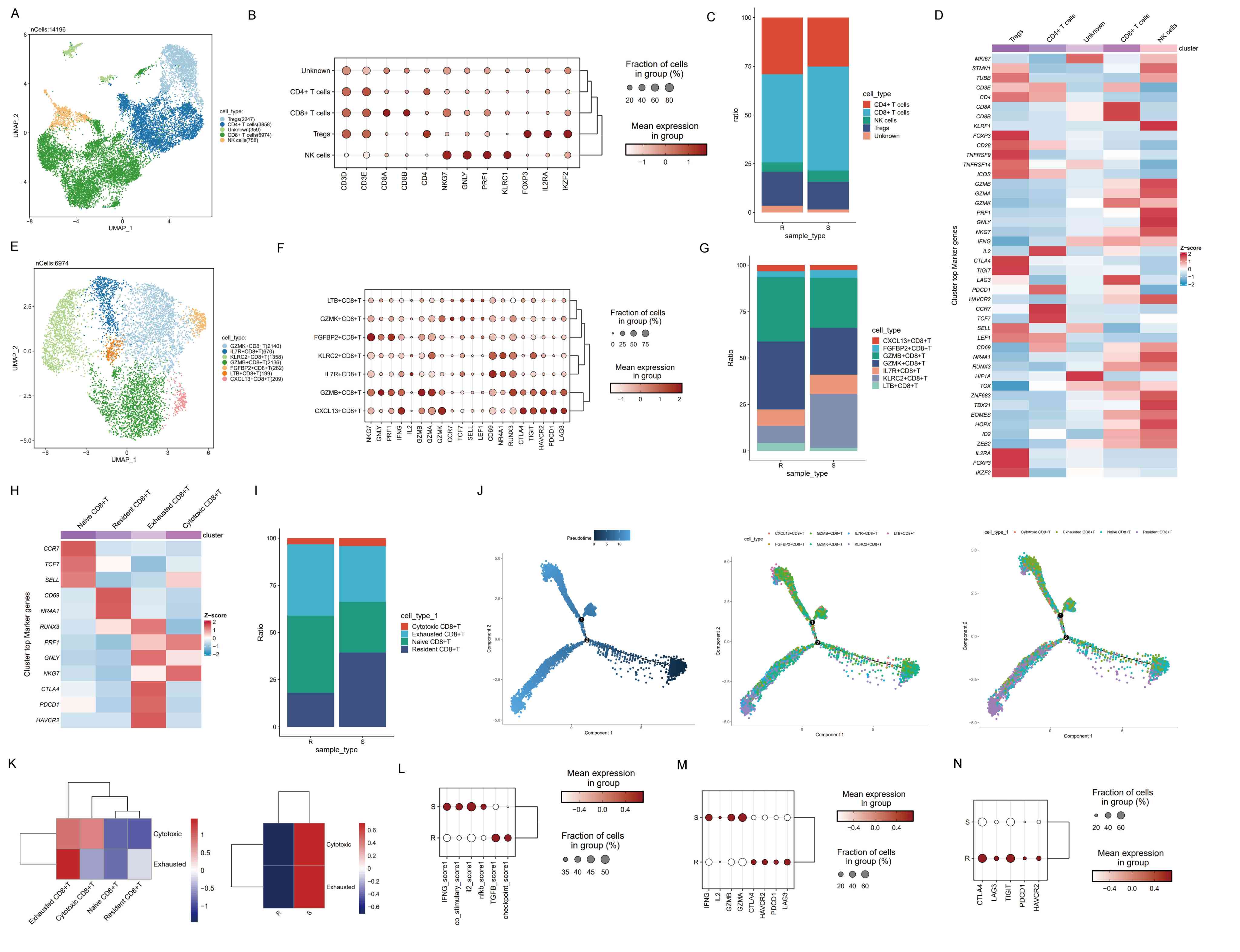


**Figure S2.** T/NK reprograming analysis. (A) UMAP plot of T cells population. (B) Dot plot showing expression of marker genes of different T/NK cell types. (C) Bar plot of cell type proportion shown as fractions of total T/NK cells in different sample types. (D) Heatmap showing the expression of canonical marker genes for T/NK cells subtypes. (E) UMAP plot of CD8+ T cells, colored by distinct subclusters. (F) Dot plot showing the expression of canonical marker genes across each CD8+T cell subcluster. (G) Bar plot of cell type proportion of fractions for distinct subclusters of CD8+T cells in different sample types. (H) Heatmap showing the expression of canonical marker gene for defined CD8+T cell subtypes. (I) Frequency of defined CD8+ T cell subtypes in different sample types. (J) The differentiation trajectory of CD8+ T cells, plot by pseudo-time (left), subclusters (middle) and defined subtypes (right). (K) Comparisons between the average level of cytotoxic and exhausted signature scores for different CD8+ T cells subtypes (left) and different sample types (right). (L) Dot plot showing the expression levels of cytotoxic and inhibitory genes in CD8+T cells. (M) Enrichment scores of T cell activated and suppressive pathways in CD8 + T cells. (N) Inhibitory genes expression levels in Tregs of different sample types.


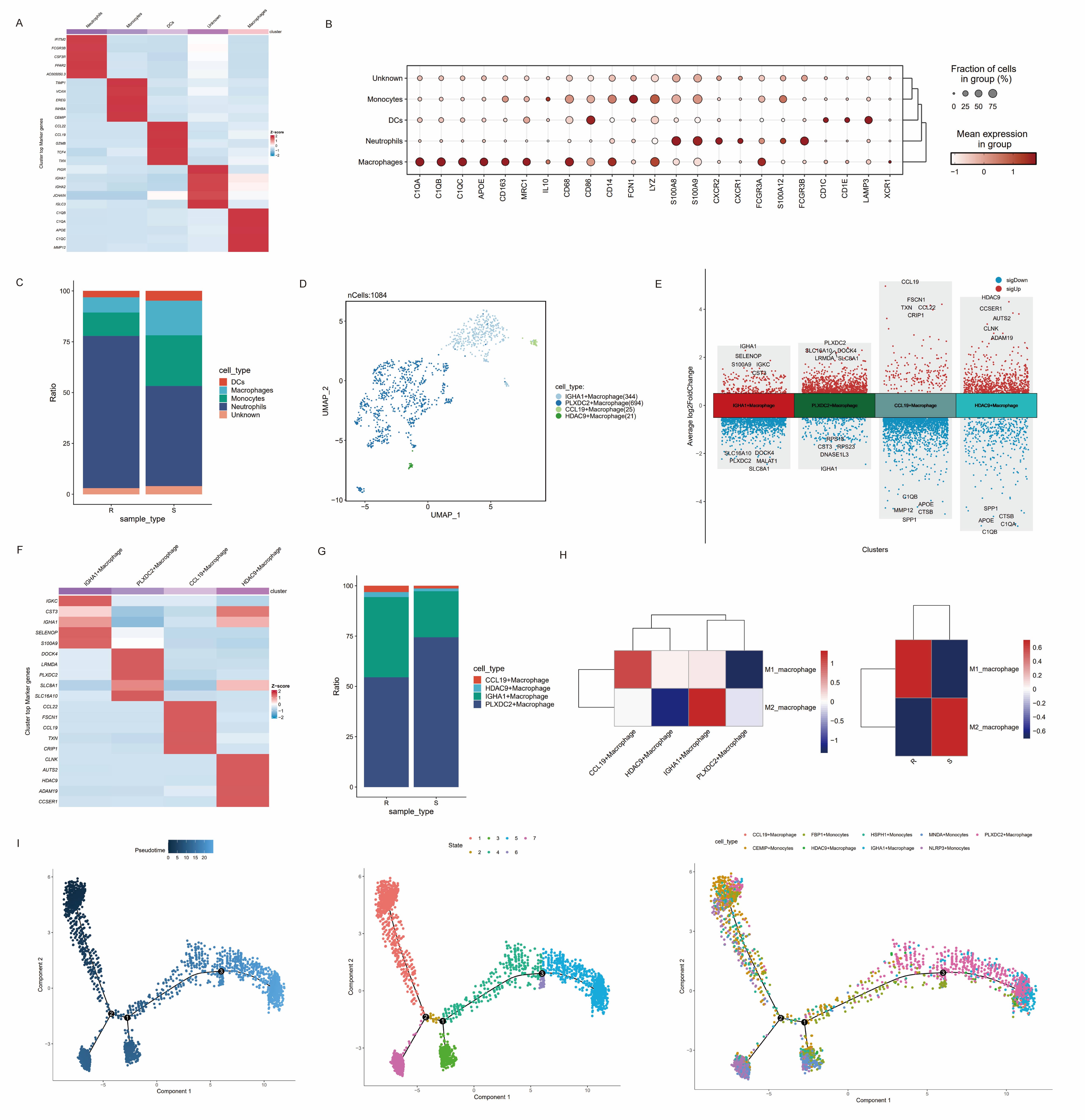


**Figure S3.** Macrophage reprograming analysis. (A) Heatmap of top marker genes for different subtypes of myeloid cells. (B) Dot plot showing expression of marker genes for different subtypes of myeloid cells. (C) Bar plot of cell type proportion for myeloid cells in different sample type. (D) UMAP plot of macrophage subtypes. (E) Volcano plot showing top marker genes of each macrophage subtype. (F) Heatmap showing the expression of marker genes across each macrophage subtype. (G) Frequency of macrophage subtypes in different sample types. (H) Comparisons between the signature score for M1 and M2 polarization in different macrophage subtypes and sample types. (I) The differentiation trajectory of monocytes and macrophages (colored by pseudo-time, state and cell subtypes).


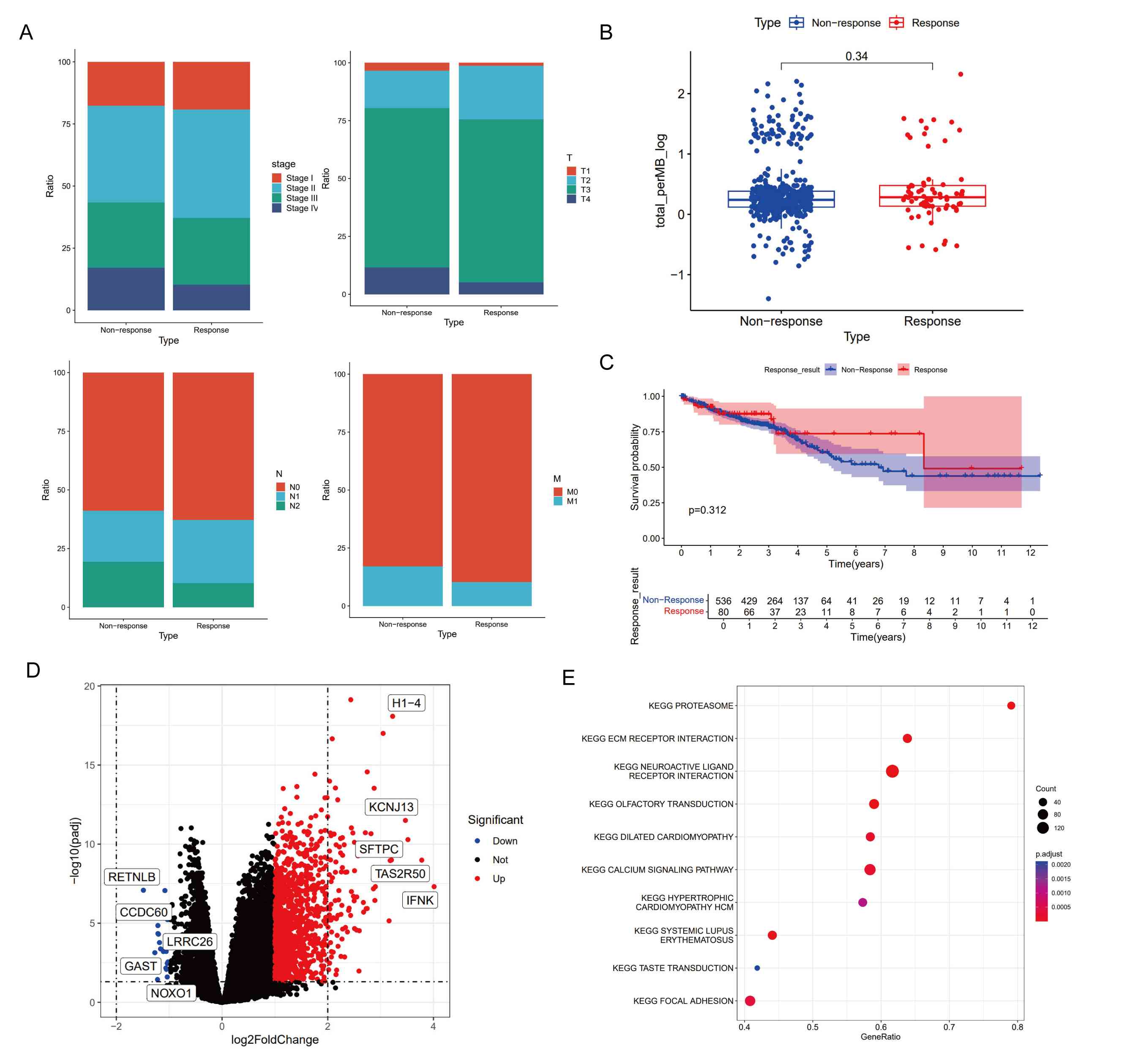


**Figure S4.** Differential analysis of potential response and non-response group. (A) The distribution of different clinicopathological features between potential responders and non-responders. (B) Boxplot showing the difference of TMB scores in different groups. (C) Survival curves showing the difference of OS in different groups. (D) Volcano plot of DEGs between different groups. (E) Dot plot demonstrating the GSEA results inferring signaling pathways enriched in non-responders.

**Table S1.** The results of scRNA-data pre-processing for each sample.

| Sample id | Estimated Number of Cells | Mean Reads per Cell | Median Genes per Cell | Number of Reads | Valid Barcodes | Sequencing Saturation | Total Genes Detected | Median UMI Counts per Cell |
| --- | --- | --- | --- | --- | --- | --- | --- | --- |
| R1 | 8626 | 45171 | 1835 | 389644290 | 94.10% | 52.00% | 31493 | 5189 |
| R2 | 12297 | 35362 | 1467 | 434845616 | 97.30% | 59.60% | 30590 | 4781 |
| R3 | 10542 | 38117 | 1968 | 401831188 | 97.70% | 61.50% | 30609 | 4640 |
| S1 | 8792 | 52102 | 1838 | 458081288 | 97.20% | 63.70% | 30265 | 7782 |
| S2 | 10415 | 40246 | 2293 | 419160347 | 97.20% | 58.20% | 32131 | 5946 |
| S3 | 9273 | 45524 | 2677 | 422147786 | 97.30% | 47.00% | 31332 | 7950 |

**Table S2.** Gene list of function signatures.

| Module/signature | Gene symbol |
| --- | --- |
| Cytotoxic signature for CD8+ T cells | GZMA, GZMB, GZMK, GNLY, IFNG, PRF1, NKG7 |
| Exhausted signature for CD8+ T cells | LAG3, TIGIT, PCCD1, HAVCR2, CTLA4, LAYN, ENTPD1 |
| IFNG genes | IFNG, CXCL9, CXCL10, STAT1, IRF1 |
| Co-stimulatory genes | CD28, ICOS, CD40LG, TNFRSF4, TNFRSF9, TNFRSF18 |
| IL2 genes | IL2, IL2RA, IL2RB, IL2RG, JAK1, JAK3, STAT5A, STAT5B, SOCS1, SOCS3 |
| NFκB genes | IKBKB, IKBKG, MAP3K7, NFKB1, RELA, NFKBIA |
| TGFB genes | TGFB1, TGFB2, TGFBR1, TGFBR2, SMAD2, SMAD3, SMAD4, FOXP3, IL10, CTLA4 |
| Checkpoint genes | PDCD1, CTLA4, LAG3, HAVCR2, CD86, CD274, LGALS9, PTPN11, CSK, BTLA |
| M0_macrophage | ACP5, BHLHE41, C5AR1, CCDC102B, CL22, CCL7, COL8A2, CSF1, CXCL3, CXCL5, CYP27A1, DCSTAMP, GPC4, HK3, IGSF6, MARCO, MMP9, NCF2, PLA2G7, PPBP, QPCT, SLAMF8, SLC12A8, TNFSF14, VNN1 |
| M1_macrophage | ACHE, ADAMDEC1, APOBEC3A, APOL3, APOL6, AQP9, ARRB1, CCL19, CCL5, CCL8, CCR7, CD38, CD40, CHI3L1, CLIC2, CXCL10, CXCL11, CXCL13, CXCL9, CYP27B1, DHX58, EBI3, GGT5, HESX1, IDO1, IFI44L, IL2RA, KIAA0754, KYNU, LAG3, LAMP3, LILRA3, LILRB2, NOD2, PLA1A, PTGIR, RASSF4, RSAD2, SIGLEC1, SLAMF1, SLC15A3, SLC2A6, SOCS1, TLR7, TLR8, TNFAIP6, TNIP3, TRPM4 |
| M2_macrophage | ADAMDEC1, AIF1, ALOX15, CCL13, CCL14, CCL18, CCL23, CCL8, CD209, CD4, CD68, CFP, CHI3L1, CLEC10A, CLEC4A, CLIC2, CRYBB1, EBI3, FAM198B, FES, FRMD4A, FZD2, GGT5, GSTT1, HRH1, HTR2B, MS4A6A, NME8, NPL, P2RY13, PDCD1LG2, RENBP, SIGLEC1, SLC15A3, TLR8, TREM2, WNT5B |
| CEMIP+ Monocytes | CEMIP, CXCL5, SOX5, SPP1, MMP1 |
| CCL4+ Neutrophils | CCL4, C15orf48, CCL3, PI3, SLAMF7 |
| MMP3+ Fibroblasts | MMP3, MMP1, CEMIP, IL11, CXCL5 |
